# Identification challenges of *Castilleja* (Orobanchaceae) on iNaturalist

**DOI:** 10.1371/journal.pone.0311088

**Published:** 2024-10-24

**Authors:** Marco Bürger, Joanne Chory

**Affiliations:** 1 Plant Biology Laboratory, Salk Institute for Biological Studies, La Jolla, California, United States of America; 2 Howard Hughes Medical Institute, Salk Institute for Biological Studies, La Jolla, California, United States of America; L3 Scientific Solutions, GERMANY

## Abstract

Citizen science platforms like iNaturalist play a crucial role in biodiversity monitoring. However, the identification of plants from the genus *Castilleja* (Indian Paintbrush), which comprises about 200 species with often similar features and frequent introgression, presents considerable challenges. Our study examines the agreement between initial identifications (often made by computer vision algorithms), subsequent identifications, and the final Research-Grade identifications for *Castilleja* species on iNaturalist. We focus on prevalent identification problems within this genus, particularly noting that *Castilleja densiflora* and *Castilleja exserta* are most frequently confused. This study highlights the need for improved algorithms to enhance initial species identification accuracy, especially for complex genera like *Castilleja*. Our findings have implications for the efficiency of the identification process on citizen science platforms and underscore the importance of expert verification in challenging taxonomic groups.

## Introduction

Citizen science has emerged as a powerful tool in ecology and conservation, enabling the collection of species observation data across broad geographical scales. Platforms like iNaturalist have revolutionized the way scientists and the public engage in documenting and understanding biodiversity [1]. The data collected through these platforms have become increasingly valuable for scientific research and decision-making, informing conservation strategies, habitat management, and restoration efforts [2]. However, the accuracy of species identifications on citizen science platforms is crucial for ensuring the quality and reliability of the data [3]. A comparison between the data quality of iNaturalist observations and digitized herbarium specimen data for flowering plant families in the southeastern United States showed that there was no significant difference between the two datasets. In fact, museum specimens were misidentified slightly more often than iNaturalist observations. This finding underscores the potential of citizen science platforms like iNaturalist to provide occurrence information with a level of accuracy comparable to traditional data sources [4]. Moreover, data validation and the potential of citizen science platforms to generate reliable biodiversity data varies depending on the taxonomic group and the level of observer expertise, as demonstrated in a report that evaluated the accuracy of iNaturalist data for selected plant and animal species in South Korea [5]. This underscores the need for expert verification and the integration of citizen science data with traditional data sources to enhance our understanding of species distributions and inform conservation efforts.

The genus *Castilleja*, commonly known as Indian Paintbrush, presents a considerable challenge for species identification. With approximately 200 species, overlapping morphological characteristics, and the occurrence of introgression, accurately identifying *Castilleja* species can be difficult even for experienced botanists [6, 7]. Incorrect or delayed identifications within this genus on citizen science platforms like iNaturalist can have substantial implications for the quality of data collected and, consequently, for research and conservation efforts that rely on these data. Investigating the patterns of these difficulties on iNaturalist is crucial for understanding the limitations of citizen science data and developing strategies to improve the accuracy of species identifications. In this study, we analyze the agreement between initial and subsequent identifications and the final Research-Grade identifications of *Castilleja* species on iNaturalist and identify the most common species involved in disagreements, both within and outside the genus. By examining the geographical overlaps between species and their misidentifications, we seek to provide insights into the factors contributing to these errors and suggest potential improvements to the iNaturalist process.

The importance of timely and accurate identifications on citizen science platforms is critical for rare plant conservation, as demonstrated by three cases involving *Castilleja* species. For instance, *Castilleja puberula*, a Tier 2 Plant of Greatest Conservation Need in Colorado [8], faces challenges in monitoring due to misidentifications on iNaturalist. The platform’s algorithm often suggests the more common species *Castilleja flava* or *Castilleja occidentalis*, resulting in many observations not reaching Research-Grade status. This uncertainty hampers our understanding of *C*. *puberula*’s population size, potentially undermining conservation efforts. Similarly, *Castilleja christii*, one of Idaho’s rarest plants [9], known from a single population, is a candidate for the Endangered Species Act. Since 2021, only 10 of 31 observations on iNaturalist have achieved Research-Grade status, with frequent misidentifications as more common *Castilleja* species. Such inaccuracies could severely impede conservation efforts for this critically endangered endemic. Finally, *Castilleja ambigua* var. *heckardii* was previously identified as a different subspecies or dismissed as a hybrid. However, it is now recognized as a rare, localized endemic [10]. Delays in accurate identification on platforms like iNaturalist can skew our understanding of its distribution and abundance and delay the protection of its observations.

These cases highlight the necessity of a comprehensive analysis of *Castilleja* identifications on citizen science platforms. By comparing initial and subsequent identifications to final Research Grade determinations, we aim to pinpoint the most significant challenges in *Castilleja* species recognition. This analysis will identify which species are most difficult to distinguish initially, providing insights to enhance the identification process and improve the reliability of citizen science data for rare species conservation.

## Methods

### Data collection and processing

The R software environment version 4.1.1 [11] was used for all data analysis and visualization. *Castilleja* species data were loaded from a CSV file using the ‘readr’ package [12]. The iNaturalist API was queried using the ‘httr’ package [13] to fetch observation data for each species, specifying taxon ID and quality grade ("research"). The API response was parsed using ‘httr’, and the total number of results and observation data were extracted. For each observation, the complete history log of identifications was retrieved to analyze the progression of species identification. This process involved downloading and extracting all identification entries associated with each observation. Any identification entry that deviated from the final, community-confirmed research-grade identification was flagged. Nomenclatural adjustments, such as taxon splits, reassignments, or differing flora treatments, were excluded from the incorrect identifications count. The function returned a data frame containing the observation ID, a Boolean value indicating whether the species was identified correctly according to the final Research-Grade entry, the correct species ID, any incorrect species IDs or other taxon IDs, latitude and longitude coordinates, and whether computer vision was used for the initial identification.

### Identification analysis

The analysis of identification frequencies for *Castilleja* species was performed using the ‘dplyr’ [14], ‘readr’, ‘stringr’ [15], ‘tidyr’ [16], and ‘tidytext’ [17] packages in R. Identification data and taxon information were loaded from CSV files. A named vector was created to map taxon IDs to species names, and a function was defined to map taxon IDs to species names from the taxon data. Functions were created to determine the affected species and their names for each identification. The identification data was then processed to prepare a table of identification frequencies. The ‘unnest_tokens’ function from the ‘tidytext’ package was used to split the identification IDs into individual words, and the frequency of each identification was calculated. A function was defined to sum up the counts for each identification ID and provide a breakdown by species. The identification frequency table was modified to include the total counts and counts breakdown by species using the ‘dplyr’ package [14].

### Geographical overlap analysis

The analysis of geographical range overlap and identifications for *Castilleja* species on iNaturalist was performed using the ‘ggplot2’ [18], ‘sf’ [19, 20], ‘dplyr’, ‘readr’, ‘rnaturalearth’ [21], ‘stringr’, and ‘rgeos’ [22] packages in R. Taxa data and species identification data were loaded from CSV files. Functions were defined to convert the data to spatial data frames, generate convex hulls for sets of points, and check if points are within a polygon. The ‘generate_maps_for_pairs’ function was defined to generate maps for each pair of taxa and create an output table. This function filtered the observation data for the specified taxa, converted the data to spatial data frames, created convex hulls for each taxon, checked if observations were inside the convex hulls, and calculated the counts of observations inside each hull and their overlap. The ‘lapply’ function was used to apply the ‘generate_maps_for_pairs’ function to each row of the ‘pairs_for_maps’ data frame, generating maps and summary data for each pair of taxa. The summary data frames were combined using ‘do.call’ and ‘rbind’.

## Results

We analyzed a total of 105,218 *Castilleja* observations on iNaturalist and examined each observation’s history log to assess the difference between initial and subsequent and the final Research-Grade identifications.

**Fig 1:**
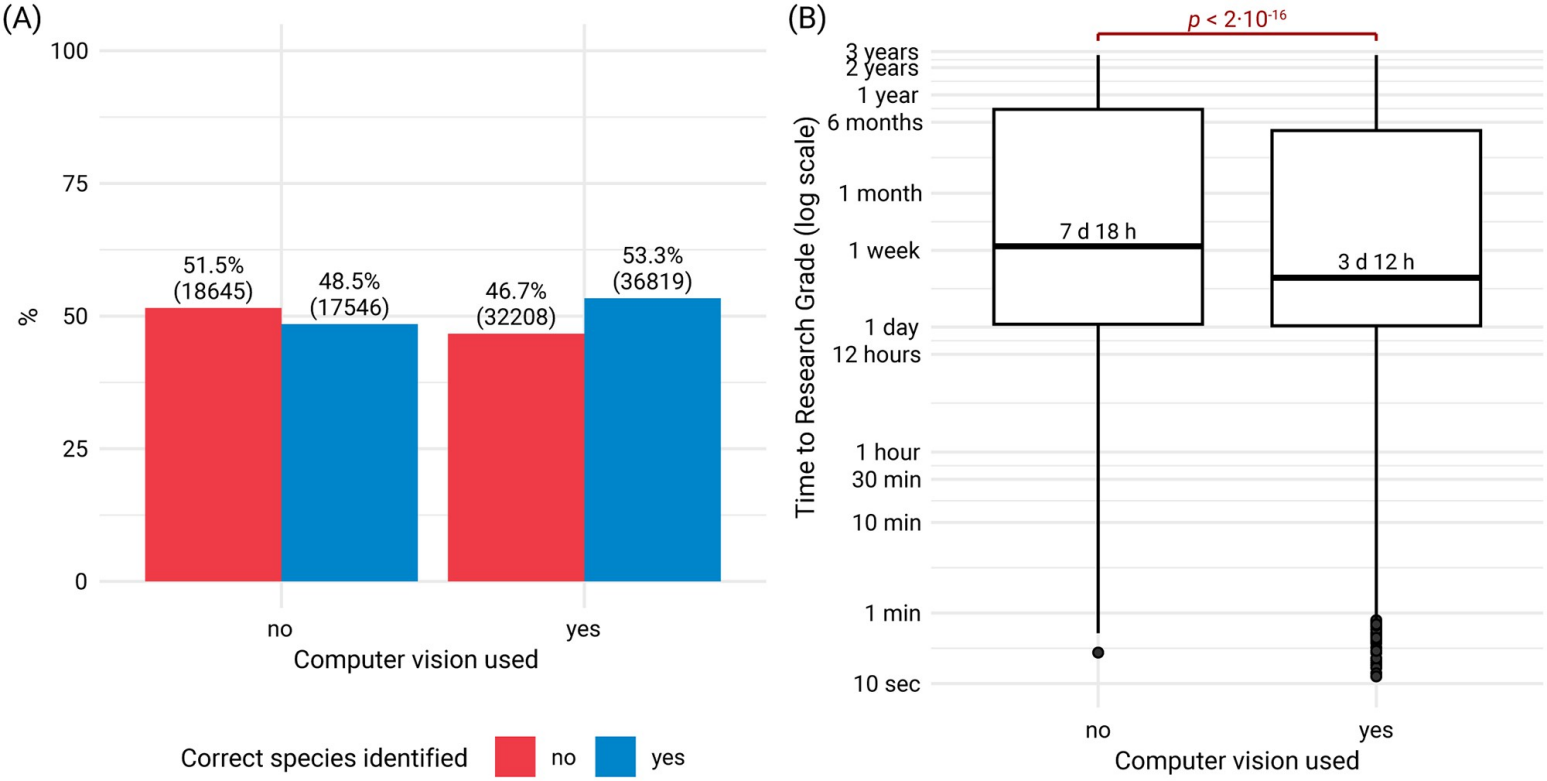
Impact of computer vision on species identification accuracy and time to Research Grade for Castilleja observations. **(A)** Percentage of species identifications with and without computer vision assistance. Bars show the proportion of initial identifications matching the later Research-Grade identifications. Numbers in parentheses indicate the count of observations in each category. **(B)** Box plot showing the distribution of time taken for observations to reach Research-Grade status, comparing observations with and without computer vision assistance. Box boundaries represent the first and third quartiles, with the median indicated by the bold line. Whiskers extend to 1.5 times the interquartile range, and points beyond represent outliers. Median times are labeled for each group.

We investigated possible differences in species identification accuracy and time to Research-Grade status between observations with and without computer vision assistance. When computer vision was used for the initial identification, 53.3% of these matched with the later identification at Research Grade level, compared to 48.5% when computer vision was not used (Fig 1A). The time to reach Research Grade was significantly shorter with computer vision (median 3.5 days) than without (median 7 days 18 hours) (Fig 1B). Additionally, the distribution of time to Research-Grade status was highly skewed, with means considerably higher than medians in both groups (4 months 3 days with computer vision; 5 months 10 days without). This suggests that while computer vision generally accelerates the identification process, a subset of observations in both groups required substantially more time to reach Research Grade.

**Fig 2:**
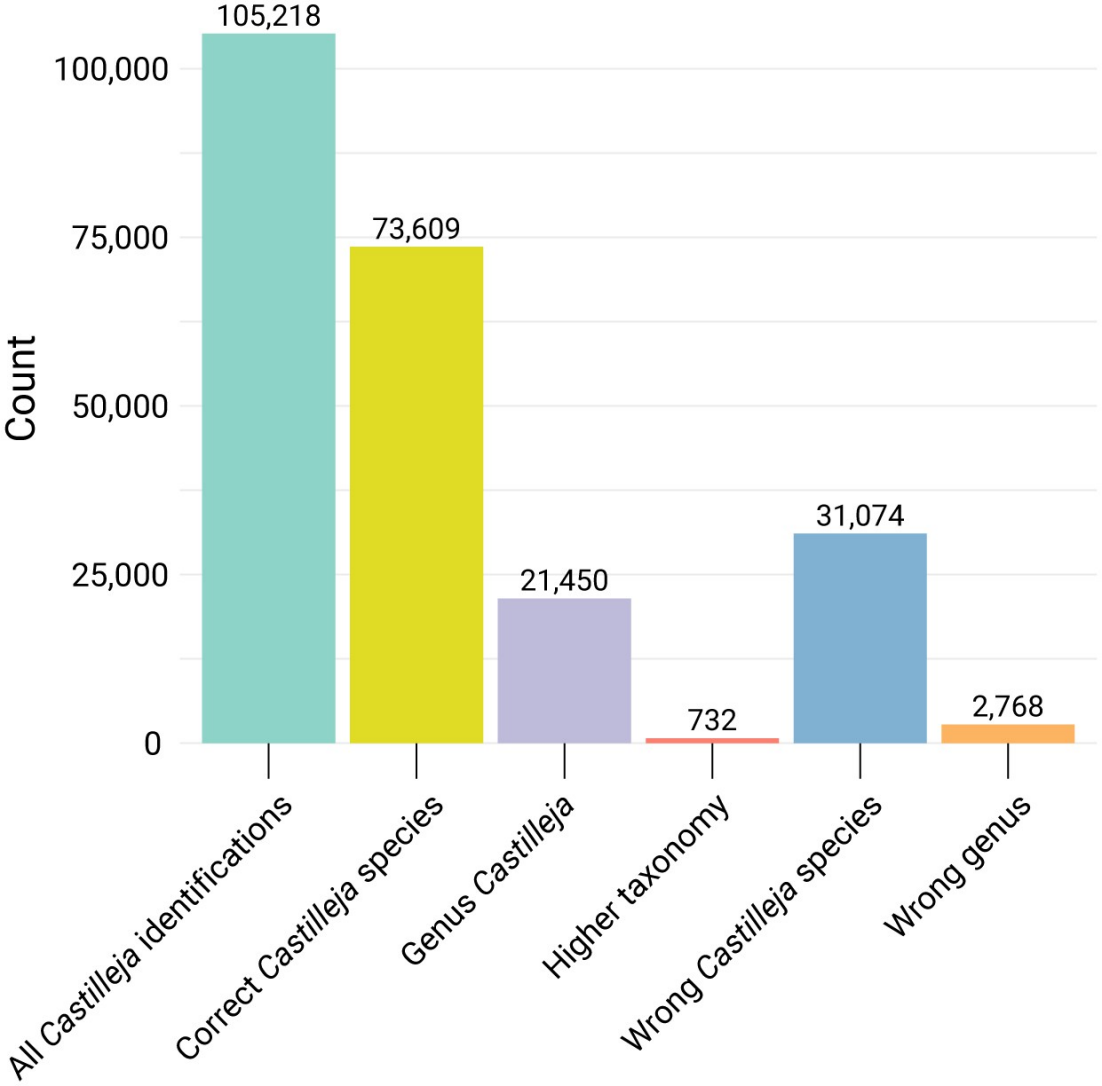
Identification statistics for *Castilleja* species observations. The chart was created using R, with the ’ggplot2’ and ’tidyr’ packages for data visualization and manipulation, respectively.

**Fig 3:**
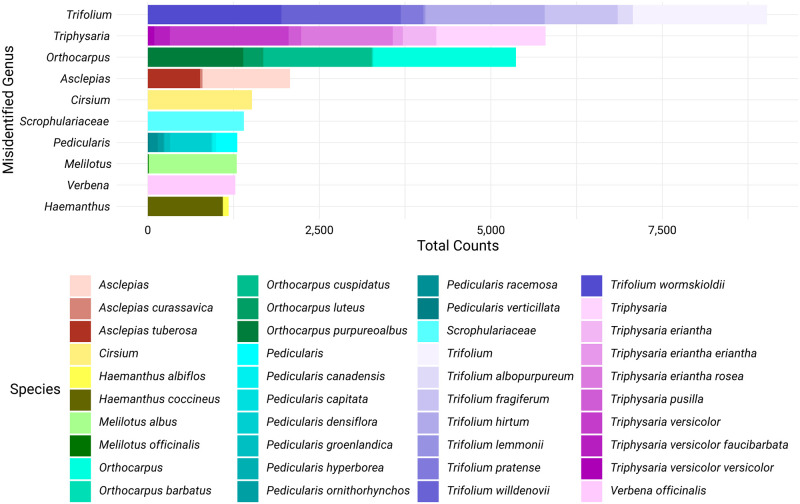
Distribution of incorrect *Castilleja* identifications across the top 10 misidentified genera, with a breakdown of the most frequently misidentified species within each genus. The data were processed using the R packages ‘dplyr’ for data manipulation, ‘ggplot2’ for visualization, ‘readr’ for reading the CSV file, ‘stringr’ for string manipulation, and ‘forcats’ [29] for reordering factor levels. The genera are ordered by their total identification counts, and the species within each genus are represented by a color gradient.

Out of the total of 105,218 observations, 73,609 (70.0%) were identified as the correct species. 21,450 (20.3%) were identified only at the genus level as *Castilleja*, while 731 (0.7%) were correctly assigned to a higher taxonomic level, such as *Lamiales* or plant. However, 31,074 (29.5%) observations were assigned to the wrong *Castilleja* species before reaching Research Grade, and 2,768 (2.6%) were incorrectly identified as a species from a different genus (Fig 2). Further analysis of these identifications at the genus level revealed that the majority of these errors could be attributed to species within the genera *Trifolium*, *Triphysaria*, and *Orthocarpus* (Fig 3).

**Fig 4:**
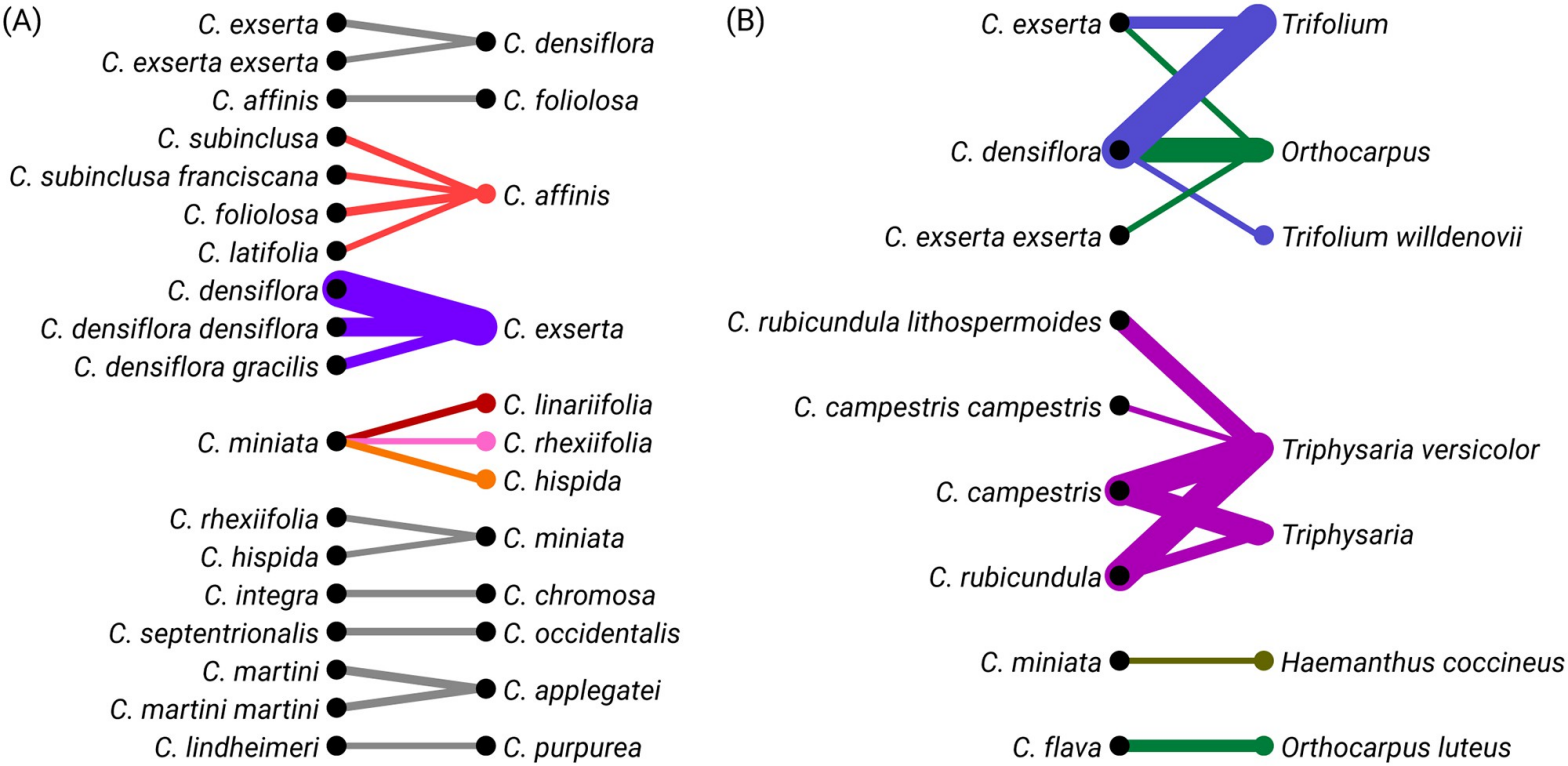
Bipartite network visualization of incorrect *Castilleja* species identifications. The data were processed using the R packages ‘dplyr’ for data manipulation, ‘readr’ for reading the CSV file, ‘igraph’ for network creation, ‘ggraph’ for network visualization, and ‘tidyr’ for data tidying. Identification data was filtered based on a minimum incorrect identification count of 50. The edges represent incorrect identifications between species or genera, with the edge width corresponding to the number of incorrect identifications.

We then investigated identification patterns within the genus *Castilleja*. The most common incorrect identification before reaching Research Grade was *Castilleja densiflora* (*C*. *densiflora*) being mistaken for *C*. *exserta*, with the reverse error occurring much less frequently. *C*. *miniata* was commonly incorrectly identified as *C*. *linarifolia*, *C*. *rhexiifolia*, or *C*. *hispida* (Fig 4A). When considering out-of-genus data, our analysis showed that *C*. *exserta* and *C*. *densiflora* were responsible for the majority of incorrect identifications involving the genera *Trifolium* and *Orthocarpus*. Similarly, *C*. *rubicundula* and *C*. *campestris* accounted for most of the incorrect assignments to the genus *Triphysaria* (Fig 4B).

**Fig 5:**
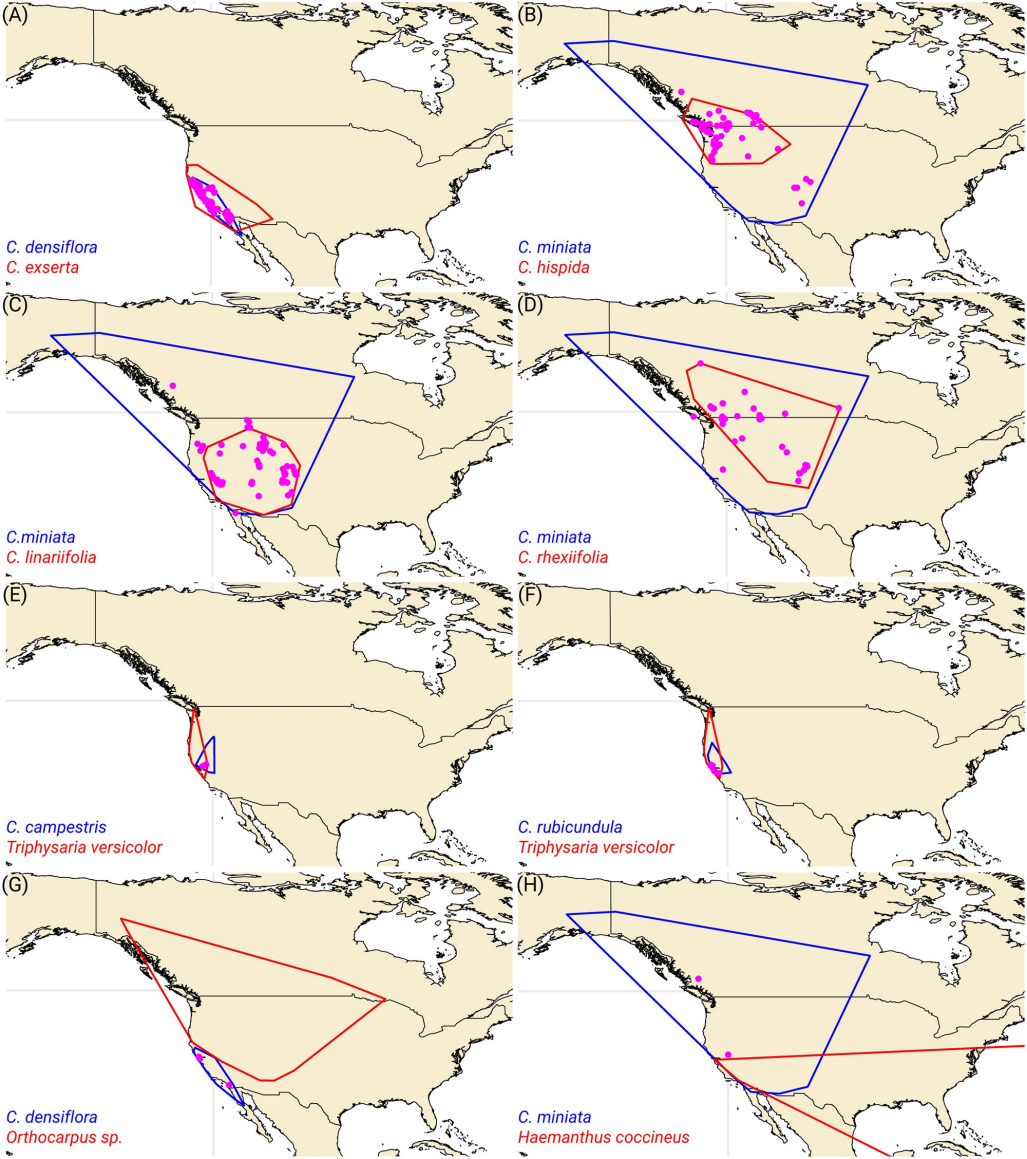
Maps displaying the range overlap and locations of incorrect identifications for various species pairs. The maps display the convex hulls of the species ranges in blue and red, with incorrect identifications marked as magenta points. The data were processed using the R packages ‘ggplot2’ for visualization, ‘sf’ for spatial data handling, ‘dplyr’ for data manipulation, ‘readr’ for reading CSV files, ‘rnaturalearth’ for world map data, ‘stringr’ for string manipulation, and ‘rgeos’ for geometric operations.

Subsequently, we examined the geographical overlaps between different *Castilleja* species and their identifications deviating from Research Grade. All incorrect identifications of *C*. *densiflora* as *C*. *exserta* occurred within the overlapping ranges of these two species, with the range of *C*. *densiflora* entirely embedded within the larger range of *C*. *exserta* (Fig 5A). Although *C*. *miniata* has a larger range than *C*. *hispida*, some of the incorrect assignments were found outside the known range of *C*. *hispida* (Fig 5B). A similar pattern was observed when examining the incorrect identifications of *C*. *miniata* as *C*. *linarifolia* (Fig 5C) or *C*. *rhexiifolia* (Fig 5D). Wrong identifications of *C*. *campestris* as *Triphysaria versicolor* were all located within the overlapping range of both species (Fig 5E), which was also the case for incorrect identifications of *C*. *rubicundula* as *T*. *versicolor* (Fig 5F). Notably, all incorrect identifications of *C*. *densiflora* as *Orthocarpus* occurred outside the known range of *Orthocarpus sp*. (Fig 5G), and all wrong identifications of *C*. *miniata* as *Haemanthus coccineus* were found outside the range of *Haemanthus coccineus* (Fig 5H).

## Discussion

The iNaturalist algorithm employs a multi-faceted approach to suggest species identifications, primarily relying on visual similarity and geographic proximity. While this strategy proves effective for many species, it falls short when dealing with visually similar species that share overlapping geographical ranges, such as *C*. *densiflora*, *C*. *exserta*, and *Trifolium*. The high number of identifications diverging from the final Research-Grade identification among these species highlights some of the limitations of the current algorithm and underscores the need for a more nuanced approach to species identification.

A comprehensive study about the iNaturalist platform reported that the median time to reach Research Grade across all iNaturalist observations had decreased to just above 4 hours by 2021 [23]. However, in our study of *Castilleja*, the median time was much longer, with initial observations assisted by computer vision taking 3.5 days and those without taking over 7 days to reach Research-Grade status. This discrepancy likely reflects the complexity of distinguishing species within this genus, where subtle morphological differences complicate the identification process. While computer vision can speed up identification in general, its performance decreases with visually similar taxa like *Castilleja*. This suggests that species complexity, expert input availability, and image quality may play larger roles in certain taxa. In our study, we focused on observations that had reached Research-Grade status, thus excluding unreviewed observations. Additionally, while Research-Grade status is generally reliable, errors may occur when initial incorrect identifications are confirmed by one or by multiple users. Given the complexity of distinguishing *Castilleja* species from photographs alone and the volume of observations, we did not revalidate these identifications.

The challenge of accurately identifying species with subtle morphological differences is not unique to iNaturalist. Certain plant families, such as Orchidaceae and Brassicaceae, are more challenging to identify accurately due to their high species diversity and visual similarity when using the Pl@ntNet platform [24], and certain species groups, such as gulls and sparrows, are more prone to misidentification on the eBird platform due to their visual similarity [25].

Our study on *Castilleja* identification patterns on iNaturalist complements these previous studies by providing a detailed analysis of the factors influencing identification accuracy within this specific genus. To improve the accuracy of the iNaturalist algorithm, particularly for complex genera like *Castilleja*, we propose the development of a specialized training set that incorporates user input on distinctive morphological features. By allowing experienced users to highlight or circle specific parts of the plant that are crucial for distinguishing between similar species, the algorithm could learn to prioritize these features in the identification process. For example, the position of the stigma is a key diagnostic characteristic for differentiating between *C*. *densiflora* and *C*. *exserta*. If experienced users consistently highlight this feature, the algorithm could assign greater weight to it when suggesting identifications of these species for less experienced users. Alternatively, expert botanists could pre-select the most important diagnostic features for each species and weigh them in the algorithm. The algorithm could then automatically prioritize the shape or color of the bracts or stigma position over other features, leading to more reliable identifications. These approaches aligns with a previous report highlighting the importance of observer expertise and visual aids in improving the reliability of citizen science data for research and conservation purposes [26]. In addition, other studies have emphasized the role of education, engagement, and expert verification in enhancing data quality when dealing with visually similar species and the challenges posed by citizen science data [27, 28]. Although 70% of *Castilleja* observations were correctly identified immediately, the remaining 30% represent thousands of observations per year, delaying their availability as Research-Grade observations.

The development of a specialized training set and the technical implementation on the iNaturalist code level would require collaboration between iNaturalist developers, botanists, and the broader user community. However, the potential benefits of these improvements are substantial. By increasing speed and accuracy of species-level data, iNaturalist could provide a more reliable resource for researchers, conservation practitioners, and decision-makers. This, in turn, could lead to better-informed conservation strategies, habitat management decisions, and ecological research outcomes.
